# CDX2 expression is concordant between primary colorectal cancer lesions and corresponding liver metastases independent of chemotherapy: a single-center retrospective study in Japan

**DOI:** 10.18632/oncotarget.24842

**Published:** 2018-03-30

**Authors:** Yasuyuki Shigematsu, Kentaro Inamura, Yoshihiro Mise, Akio Saiura, Emil Rehnberg, Noriko Yamamoto, Yuichi Ishikawa, Shunji Takahashi, Hiroaki Kanda

**Affiliations:** ^1^ Department of Pathology, The Cancer Institute of Japanese Foundation for Cancer Research (JFCR), Koto, Tokyo 135-8550, Japan; ^2^ Division of Gastroenterology Center, The Cancer Institute Hospital, JFCR, Koto, Tokyo 135-8550, Japan; ^3^ Division of Business Intelligence, Labs BI, Shanghai 200040, China; ^4^ Division of General Oncology, The Cancer Institute Hospital, JFCR, Koto, Tokyo 135-8550, Japan

**Keywords:** CDX2, heterogeneity, colorectal cancer, chemotherapy, liver metastasis

## Abstract

**Objective:**

Loss of caudal-type homeobox transcription factor 2 (CDX2) expression in colorectal cancers (CRCs) has recently been proposed as a promising predictive biomarker for not only prognosis but also response to chemotherapy. However, the relationship between alterations in CDX2 expression during cancer progression and response to chemotherapy remains unclear. We herein aimed to determine the concordance of CDX2 expression between primary CRCs and corresponding liver metastases, in association with chemotherapy.

**Results:**

Primary CRCs exhibited heterogeneous CDX2 expression. Seven of the 144 CRCs in the cohort (4.9%, 95% confidential interval, 2.0%–9.8%) were CDX2-negative. The concordance rate of the CDX2 expression status in patients who did not receive chemotherapy was 100% (*P* = 0.041), whereas the concordance rate among patients who received chemotherapy only after primary resection was 96.3% (*P* = 0.005). Moreover, the concordance rate in patients who received chemotherapy before both primary resection and liver metastasectomy was 100% (*P* < 0.001).

**Conclusion:**

CDX2 expression status was highly concordant between primary CRCs and corresponding liver metastases, independent of chemotherapy, suggesting that the CDX2 expression status in CRCs was not affected by metastasis or chemotherapy.

**Methods:**

A total of 144 consecutive patients with CRC who were treated at a single center in Japan between 2006 and 2014 were included. Formalin-fixed paraffin-embedded whole sections of surgically resected primary CRCs and corresponding liver metastases were assessed for CDX2 expression by immunohistochemistry.

## INTRODUCTION

Caudal-type homeobox transcription factor 2 (CDX2) regulates gut epithelial development and maturation [1–4] and is expressed within nuclei of intestinal epithelial cells from proximal duodenum to distal rectum [5]. Increased CDX2 expression, which is observed in approximately 90%–95% of colorectal adenocarcinomas [6, 7], is considered to be a highly sensitive and specific diagnostic marker for adenocarcinomas of intestinal origin [8–10]. In addition to its diagnostic utility, CDX2 is reported to exert tumor-suppressor functions by controlling a number of genes involved in proliferation, migration, and carcinogenesis [11]. However, alterations in the CDX2 expression during cancer progression have not been examined extensively. The possibility remains that genetic diversification and clonal selection might result in changes in CDX2 expression status [12–15].

Several retrospective studies suggested that the loss of CDX2 expression in colorectal cancers (CRCs) was associated with worse prognosis as well as several adverse prognostic variables, such as high histologic grade, *BRAF* mutations, and CpG island methylator phenotype positivity [16–18]. Interestingly, in early-stage CRCs, adjuvant chemotherapy could decrease recurrence risk in patients with loss of CDX2 expression, suggesting that this phenotype might be a potential predictive marker for response to chemotherapy [16]. However, in metastatic CRCs, the loss of CDX2 expression was reported to be associated with lower chemotherapy efficacy [19]. Therefore, it remains unclear whether the CDX2 expression status represents a chemosensitive phenotype.

Chemotherapy plays an important role in the treatment of CRCs. Patients with technically unresectable metastatic CRCs benefit from chemotherapy, which was shown to shift the clinical stage toward a technically resectable condition [20]. Patients with stage III and high-risk stage II CRCs also benefit from adjuvant chemotherapy, as shown by decreased risk of recurrence [21]. In this context, the impact of chemotherapy on CDX2 expression status, which is considered as a promising prognostic marker in CRCs, remains poorly understood, although the expressions of several genes were previously shown to be affected by chemotherapy [12, 22].

There is a concern about the presence or absence of heterogeneity of CDX2 expression in CRCs because there are few reports regarding CDX2 expression heterogeneity, and the previous studies [16–18] were conducted using tissue microarrays (TMAs), whose results can be affected by the heterogeneity of the target protein expression. To establish the presence or absence of the heterogeneity of CDX2 expression in CRCs, immunohistochemistry (IHC) using whole sections is necessary.

In the present study, using whole sections of lesions, we aimed to evaluate alterations in CDX2 expression during cancer progression and to assess the impact of chemotherapy on CDX2 expression in primary CRCs and corresponding liver metastases.

## RESULTS

### Patient and tumor characteristics

Demographic and clinicopathological characteristics of all CRC patients are summarized in Table 1. Briefly, at diagnosis, mean patient age was 60.1 years, and most of the patients were male (68.5%). A majority of the patients (80.5%) had liver metastases at diagnosis, and there was a median of four metastatic liver tumors (These data were not included in Table 1, and partly included in Table 2). Additionally, a majority of the primary CRCs (96.5%) were well or moderately differentiated by histological assessment. A total of 115 patients (79.9%) had left-sided CRCs. In the present study, 120 out of 144 patients (83.3%) received chemotherapy; among these, 82 patients (68.3%) received chemotherapy only before metastasectomy, whereas the remaining patients (31.7%) received chemotherapy before both primary resection and liver metastasectomy.

**Table 1:** Patients and tumor characteristics

Characteristics	Case No.
All cases	144
Mean age at metastasectomy (years)	60.1
Sex	
Male	98
Female	46
TNM stage	
I	1
II	4
III	23
IV	116
Tumor grade	
well	48
mod	91
por	5
Tumor location	
Cecum	6
Ascending	12
Transverse	11
Descending	6
Sigmoid	51
Rectum	58
Chemotherapy	
Yes	120
Before metastasectomy only	82
Before both primary resection and metastasectomy	38
No	24

**Table 2:** Relationship between clinicopathological characteristics and CDX2 expression in primary colorectal cancers

Characteristics	CDX2 expression		*P*-value
	Positive	Negative	
All cases	137	7	
Age			
≤65	97	5	1.00
65<	40	2	
Sex			
Female	45	1	0.43
Male	92	6	
Histology			
well	47	1	0.02
mod	87	4	
por	3	2	
Number of liver metastases			
<5	84	3	0.44
5≤	53	4	
Clinical stage			
I	1	0	0.23
II	4	0	
III	20	3	
IV	112	4	

### Distribution of CDX2 expression in a whole section of primary CRCs and a cut-off value to determine the CDX2 expression status

**Figure 1:**
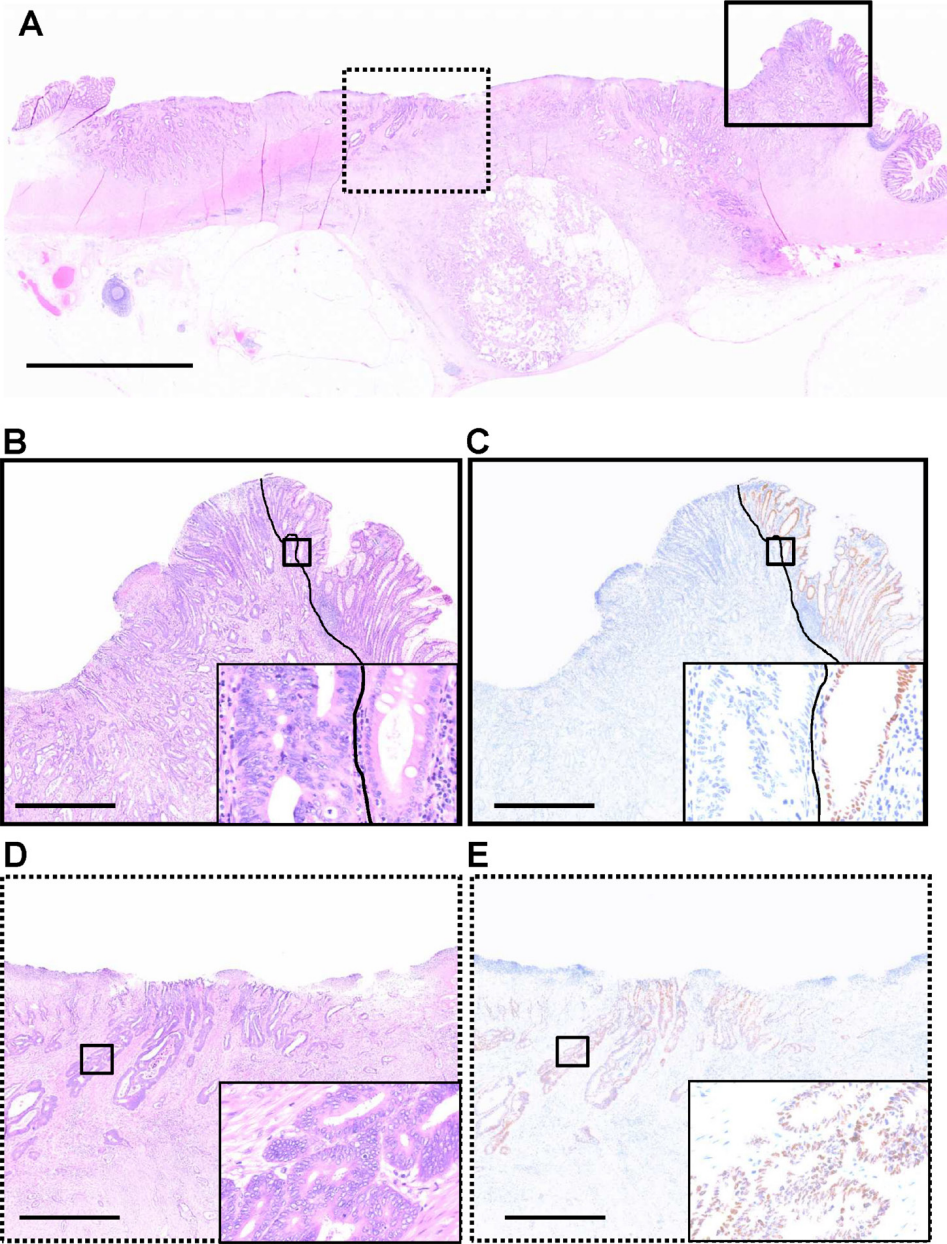
CDX2 expression in a whole formalin-fixed, paraffin-embedded section of a colorectal cancer case, which exhibits heterogeneous CDX2 expression (**A**) A representative section stained with hematoxylin and eosin. (**B**, **C**) Magnification of the area boxed by the solid line in A. A Solid line represents the boundary between cancerous (left side) and non-cancerous (right side) regions. Insets show higher magnification of areas boxed by thick solid lines. (B) Hematoxylin and eosin staining. (C) Immunohistochemistry (IHC) for CDX2. In the non-cancerous region, colonic epithelial cells with nuclear CDX2 expression serve as positive controls for CDX2 IHC. In contrast, tumor cells in the same region do not express the CDX2. (**D**, **E**) Magnification of the area boxed by the dashed line in A. Insets show higher magnification of areas boxed by thick solid lines. (D) Hematoxylin and eosin staining. (E) IHC for CDX2. In the cancerous region, cancer cells exhibit nuclear CDX2 expression. Scale bars, 5 mm (for A) and 1 mm (for B, C, D, and E).

**Figure 2:**
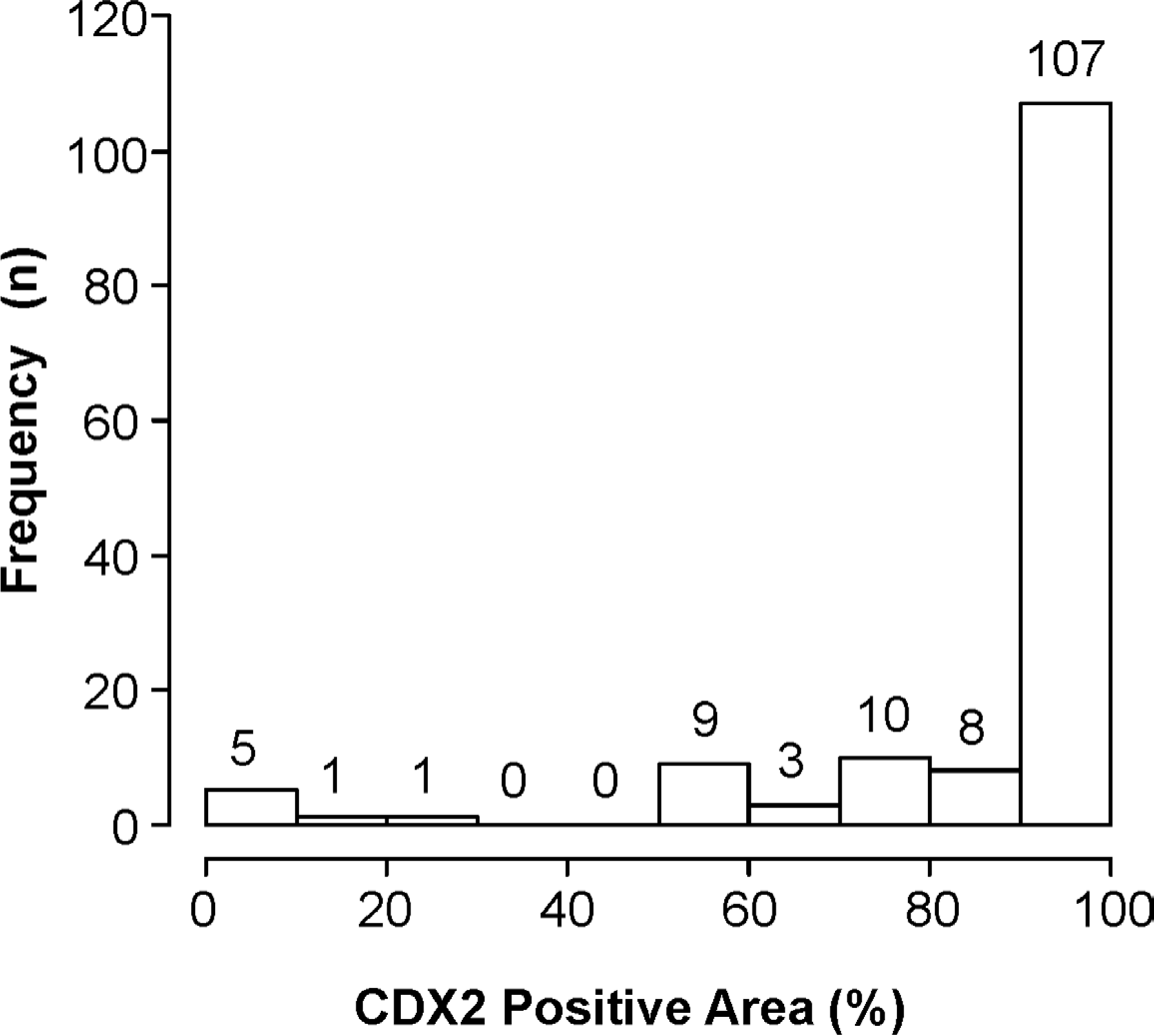
Histogram depicting the ratios of CDX2-positive cells to all cancer cells in whole sections of primary colorectal cancers In the study cohort, more than 32 cases (22.2%) showed heterogeneous CDX2 expression.

To evaluate the expression of CDX2 in CRCs, we performed IHC for CDX2 using whole sections of surgical samples (Figure 1). More than 20% of primary CRCs showed heterogeneous CDX2 expression (Figure 2). To ensure that the clinical judgment used to determine the CDX2 status of specimens was reproducible, the presence of 50% CDX2-positive cells within a whole tumor specimen was used as the cut-off point. Specifically, samples were considered as CDX2-positive if 50% or more of the cancer cells displayed widespread nuclear CDX2 expression, whereas samples were considered as CDX2-negative if less than 50% of the cancer cells showed nuclear CDX2 expression. To assess the robustness of the criterion, concordance between the CDX2 scoring results obtained by two independent investigators were assessed, which revealed a concordance rate of 97.9% (95% confidence interval [CI], 95.5%–99.2%; kappa index, 0.82), indicating that the judgment using the criterion was robust.

### Clinicopathological characteristics of CDX2-negative CRCs

Primary CRCs were categorized into CDX2-positive and CDX2-negative groups using the defined criteria (Table 2). Therefore, there were seven CDX2-negative CRCs (4.9%, 95% CI, 2.0%–9.8%) and 137 CDX2-positive CRCs in the study cohort (95.1%, 95% CI, 90.2%–98.0%). Two of the CDX2-negative primary CRCs (28.5%) were poorly differentiated adenocarcinomas, compared with three poorly differentiated primary CRCs (2.2%) in the CDX2-positive group (*P* = 0.02), confirming the previous studies reporting that CDX2-negative CRCs were more likely to be poorly differentiated [18, 23]. In contrast, there were no significant differences in age, sex, the number of liver metastases, or clinical-stage between the CDX2-positive and CDX2-negative groups.

### Concordance analysis of the CDX2 expression status between primary CRCs and corresponding liver metastases

To analyze whether the CDX2 expression status might be affected during the metastatic process, the CDX2 expression status of primary CRCs were compared with that of the corresponding liver metastases before chemotherapy (Table 3, patients without chemotherapy). There were 23 CDX2-positive and 1 CDX2-negative primary CRCs. The concordance rate of the CDX2 expression status was 100%, and the association between the CDX2 expression status of metastatic lesions and that of the primary CRC lesions was statistically significant (*P* = 0.041), indicating that the metastatic process did not affect the CDX2 expression status in CRCs.

**Table 3:** Concordance of CDX2 expression between primary colorectal cancers and corresponding liver metastases in patients with and without chemotherapy

Patients without chemotherapy				*P*-value
	Metastatic site			0.041
Primary site	Positive	Negative	total	
Positive	23	0	23	
Negative	0	1	1	
total	23	1	24	
Patients who received chemotherapy before metastasectomy only				
	Metastatic site			0.005
Primary site	Positive	Negative	total	
Positive	77	2	79	
Negative	1	2	3	
total	78	4	82	
Patients who received chemotherapy before both primary and metastatic resections				
	Metastatic site			
Primary site	Positive	Negative	total	<0.001
Positive	35	0	35	
Negative	0	3	3	
total	35	3	38	

Using samples from patients who received chemotherapy only after primary resection, we compared the CDX2 expression status between primary CRCs and corresponding metastatic lesions (Table 3, patients with chemotherapy only before metastasectomy). Among a total of 79 CDX2-positive CRCs, the change to CDX2 negativity was found in only two cases. Conversely, the metastatic lesion in one of the three patients with CDX2-negative primary CRCs that received chemotherapy was CDX2-positive. The concordance rate of the CDX2 expression status was 96.3%, and there was a significant association between the CDX2 expression status of the primary CRCs and metastatic lesions (*P* = 0.005). Therefore, the impact of chemotherapy on CDX2 expression status was considered to be minimal.

The CDX2 expression status of primary CRCs and corresponding metastatic lesions in patients who received chemotherapy before both primary resection and metastasectomy was analyzed (Table 3, patients with chemotherapy before both primary resection and metastasectomy). There were 35 CDX2-positive and 3 CDX2-negative primary CRCs. The concordance rate of the CDX2 expression status was 100%, and there was a significant association in the CDX2 expression status between primary and metastatic lesions among patients receiving chemotherapy (*P* = 0.0002). These data indicated that the CDX2 expression status was not affected by the metastatic process and chemotherapy.

To confirm that the high concordance can be achieved regardless of the cutoff point, we used a cutoff point of 20%; the numbers of CDX2-positive CRC and CDX2-negative CRC were 115 and 29, respectively, and the overall concordance rate was 95.1%, indicating high concordance (Supplementary Table 1). Therefore, we can conclude that the CDX2 expression status of CRCs is consistent between primary and liver metastatic sites regardless of the cutoff point.

### Recurrence-free survival and overall survival among CDX2-positive CRCs and CDX2-negative CRCs

In an exploratory analysis, we evaluated the association between CDX2 expression and recurrence-free survival (RFS) or overall survival (OS). The analysis revealed that median RFS in the CDX2-positive group was similar to that in the CDX2-negative group (326 days vs. 319 days, *p* = 0.99) (Supplementary Figure 1A). The three-year OS in the CDX2-positive group was 78% and that in the CDX2-negative group was 57%; however, the difference was not statistically significant (*p* = 0.65) (Supplementary Figure 1B).

### Concordance analysis of CK7, CK20, CEA, and MUC2 expression status between primary CRCs and corresponding liver metastases

As a secondary analysis, we investigated the concordance rate of cytokeratin 7 (CK7), CK20, carcinoembryonic antigen (CEA), and mucin 2 (MUC2) expression status. In the same manner as the CDX2 investigation, we used a whole-section of all the samples and set a cutoff point of 50% of tumor cells to assess whether a tumor is positive or negative for the protein expression. In the primary CRCs, the positivity rates of CK7, CK20, CEA, and MUC2 were 5%, 89%, 85%, and 3%, respectively. The overall concordance rates of the expression of CK7, CK20, CEA, and MUC2 proteins were 97%, 88%, 84%, and 98%, respectively (Supplementary Table 2). In patients without chemotherapy (*n* = 23), the concordance rates of the expression of CK7, CK20, CEA, and MUC2 proteins were 96%, 87%, 91%, and 100%, respectively. In patients who received chemotherapy before metastasectomy only (*n* = 76), the concordance rates of the expression of CK7, CK20, CEA, and MUC2 proteins were 97%, 92%, 86%, and 97%, respectively. In patients who received chemotherapy before both primary and metastatic resections (*n* = 35), the concordance rates of the expression of CK7, CK20, CEA, and MUC2 proteins were 97%, 83%, 77%, and 100%, respectively. Liver metastatic sites also showed high concordance rates with the primary CRCs for CK7, CK20, CEA, and MUC2 expression status.

### Expression status of CDX1 in the primary CRCs and its concordance between primary and liver metastatic sites

As a secondary analysis, we investigated CDX1 expression status in non-cancerous and cancerous tissues using 134 primary CRCs because CDX1 has high structural homology to CDX2 and plays complementary roles in the adult intestine [24], and its expression has been reported to inhibit colon cancer cell proliferation *in vitro* [25, 26]. In the primary sites, the nuclear expression of CDX1 was observed in the non-cancerous epithelium. In contrast to the non-cancerous epithelium, the CDX1 expression of cancerous tissue was usually very low or absent (Supplementary Figure 2). In the same manner as the CDX2 investigation, we set a cutoff point of 50% tumor cells to assess whether a tumor is positive or negative for CDX1 protein expression. Among the 134 primary CRCs, 7 (5.2%) were CDX1-positive CRCs and 127 (94.8%) were CDX1-negative CRCs.

As for the concordance analysis, the overall concordance rate of CDX1 expression was 96.3%. In patients without chemotherapy (*n* = 23), the concordance rate was 100%. In patients who received chemotherapy before metastasectomy only (*n* = 35), the concordance rate was 85.7%. In patients who received chemotherapy before both primary and metastatic resections (*n* = 76), the concordance rate was 100%. Because of the high concordance rates, we can conclude that CDX1 expression status of CRCs is consistent between primary and liver metastatic sites regardless of metastatic process and chemotherapy.

## DISCUSSION

The present study demonstrated the heterogeneity of CDX2 expression in whole CRC sections. Additionally, the results revealed the high concordance rate of CDX2 expression between primary CRCs and corresponding liver metastases, which was not affected by chemotherapy. To the best of our knowledge, this is the first report to investigate potential changes in CDX2 expression during CRC development and chemotherapy.

Previous reports utilized TMAs to analyze CDX2 expression in CRCs [23, 27–30]. One major concern of TMA analysis is that the small core samples may not be representative of the entire tumor [31, 32]. Our results demonstrating the heterogeneity of CDX2 expression in primary CRCs raise the possibility that previous studies reporting CDX2 expression using TMAs might not reflect the status of whole CRC sections. Conversely, an optimal cut-off value to determine the CDX2 expression status in whole CRC sections might be necessary if CDX2 expression determined by TMAs is utilized in daily medical practice as a prognostic or predictive biomarker for response to chemotherapy.

We hypothesized that the percentage of the CDX2-negative component in the primary CRC would increase during cancer evolution, based on recent studies showing that the loss of CDX2 expression was associated with worse prognosis [16, 18, 19]. However, our results demonstrated that the differences in CDX2 expression status between primary CRCs and their corresponding liver metastases were minimal. Additionally, the numbers of liver metastases were comparable between the CDX2-positive and -negative groups. These results suggested that while CDX2 might not play an important role in the development of liver metastasis of CRC, and the CDX2 expression status might be useful as a biomarker for the clinical evaluation of both primary CRC lesions and liver metastases.

The utility of CDX2 expression status as a predictive biomarker for response to chemotherapy remains controversial. In contrast to the suggested benefit of adjuvant chemotherapy for stage II and stage III CDX2-negative CRCs [16], the benefit of systemic chemotherapy was significantly lower for CDX2-negative metastatic CRCs than for CDX2-positive CRCs [19]. Generally, in tumors with heterogeneous components exhibiting distinct sensitivities to chemotherapy, the ratio of components conferring chemotherapy resistance increased during treatment [33]. In the present study, we found that the CDX2 expression status in liver metastases changed minimally from that in primary lesions, indicating that the CDX2 expression status might not be directly associated with chemotherapy sensitivity. Our retrospective study assessing the association between CDX2 expression status and chemotherapy sensitivity warrants further validation.

Our study has several limitations due mainly to its retrospective nature. First, we selected the patients who underwent primary CRC resection and liver metastasectomy in the current study. However, we believe that selection bias was negligible because the comparisons included primary CRC and metastatic samples that were obtained from the same patients, and the prevalence CDX2 expression loss of 4.9% was similar to those reported previously [16, 19]. Second, long-term storage of samples (3–10 years) might have affected CDX2 immunoreactivity. However, the year of diagnosis did not have a significant impact on the CDX2 expression (data not shown), suggesting that the storage period did not substantially affect CDX2 immunoreactivity. Third, the number of CDX2-negative CRC patients was relatively small, and information about CDX2 expression alterations in the CDX2-negative CRCs was limited. This is because CDX2-negative CRCs are rare, and only Japanese patients at a single cancer hospital were included in the present study. However, in this study, on an examination of cross-sections, primary CRC revealed heterogenous CDX2 expression, and 21.9% (30/137) of CDX2-positive primary CRCs also had CDX2-negative cancer cells in the tumor (Figure 2). Thus, the high concordance rate of the CDX2 expression status was achieved through expression consistency in both CDX2-positive and CDX2-negative components of the primary tumor. Although our primary hypothesis regarding the association between CRCs and CDX2 expression status was whether the alterations from CDX2-positive to CDX2-negative during tumor progression or chemotherapy were negligible, the investigation of the opposite situation also yields valuable information. In survival analysis, the number of patients with CDX2-negative CRCs is small, and the results might not be sufficiently powered to detect a difference. Further studies with a large sample size are necessary to elucidate the impact of chemotherapy or tumor progression in CDX2-negative CRCs and confirm our findings.

This is the first study to demonstrate that the CDX2 expression status in CRCs was highly concordant between primary CRCs and their corresponding liver metastases, independent of chemotherapy, suggesting that the CDX2 expression status in CRC was not affected by metastasis or chemotherapy.

## MATERIALS AND METHODS

### Patients and tissue samples

In this retrospective study, CRC surgical samples were obtained from 144 consecutive patients who underwent colorectal resection with extended lymph node dissection (D2 or D3) and liver resection for CRC at the Cancer Institute Hospital in Tokyo, Japan, between 2006 and 2014. Only patients with resected tumor samples available from both primary CRCs and corresponding liver metastases that were suitable for immunohistochemical analysis were included. In total, 288 samples consisting of primary CRCs and corresponding liver metastases from 144 patients were included. Clinical data and histological features included age, sex, tumor location, number of liver metastases, clinical-stage, history of chemotherapy, and tumor grade. Histological findings were determined according to the fourth edition of the World Health Organization criteria [34]. Tumor stage was determined according to the seventh edition of the tumor-node-metastasis staging manual of the American Joint Committee on Cancer [35]. Patients who received chemotherapy within six months before surgical resection were defined as those with a history of chemotherapy. Patients who did not give written informed consent were excluded from this study. Whole CRC sections were reviewed and confirmed by two of the investigators (Y.S. and K.I.). The study was approved by the committee for ethics at the Cancer Institute Hospital (No. 2016–1087).

### Immunohistochemical analysis

IHC was performed using 4-μm thick formalin-fixed paraffin-embedded CRC sections. Sections of the largest cross-sectional slice of the primary and metastatic lesions were used for the analysis. We used a primary mouse anti-human CDX2 monoclonal antibody (clone DAK-CDX2, 1:100 dilution, DAKO, Carpinteria, CA, USA) that was previously validated for diagnostic applications [9]. All samples were handled in an anonymous fashion. Tissue slides were stained using a Bond-III automatic stainer (Leica Microsystems, Buffalo Grove, IL, USA), and antigen detection was achieved using the Bond Polymer Refine detection kit (Leica Microsystems).

CRC diagnosis was confirmed by two experienced pathologists (Y.S. and K.I.) based on the microscopic examination of hematoxylin-eosin–stained slides. All cases were scored blindly by two experienced pathologists (Y.S. and K.I.) in an independent fashion. Cases that were difficult to interpret immunohistochemically were discussed with a third experienced pathologist (H.K.). Nuclear CDX2 expression was scored based on the number of CDX2-positive tumor cells.

In the secondary analysis, 268 matched samples from 134 patients used the following primary antibodies: anti-human CK7 monoclonal antibody (clone OV-TL 12/30, 1:200 dilution, DAKO, Carpinteria, CA, USA), anti-human CK20 monoclonal antibody (clone IT-Ks 20.8, 1:100 dilution, Progen Biotechnik, Heidelberg, Germany), anti-human CEA monoclonal antibody (clone COL-1, ready-to-use prediluted, Nichirei Bioscience, Tokyo, Japan), and anti-human MUC2 monoclonal antibody (clone Ccp58, 1:200 dilution, Leica Biosystems, Newcastle, UK). As for CDX1 IHC, anti-human CDX1 polyclonal antibody (HPA055196, 1:500 dilution, Atlas Antibodies AB, Stockholm, Sweden) and anti-rabbit IgG polyclonal antibody (211-005-109, Jackson ImmunoResearch Laboratories, Inc., West Grove, PA, USA) were used.

### Statistical analysis

All statistical analyses were conducted using R version 3.2.2 (R Foundation for Statistical Computing, Vienna, Austria). Fisher's exact test was used to analyze the association between primary CRCs and corresponding liver metastases based on the CDX2 expression status and to evaluate differences in relative frequencies of clinicopathological parameters between the CDX2-positive and -negative groups. Cohen's kappa index was used to assess the concordance of CDX2 expression status determined by the two investigators [36]. Differences between mean values of two groups were evaluated by the Mann–Whitney *U* test. Two-tailed *P* values of 0.05 or less were considered to be statistically significant. For the survival analysis between the patient subgroups, we used Kaplan–Meier curve and log-rank test. RFS and OS were calculated from the date of liver metastasectomy to the date of the first recurrence at any site or death.

## SUPPLEMENTARY MATERIALS FIGURES AND TABLES



## Abbreviations

CRC: colorectal cancer
CDX2: caudal-type homeobox transcription factor 2
IHC: immunohistochemistry
TMA: tissue microarray
CK: cytokeratin
MUC2: mucin 2
CEA: carcinoembryonic antigen
CDX1: caudal-type homeobox transcription factor 1
