# Supplementary material for: CDX2 expression is concordant between primary colorectal cancer lesions and corresponding liver metastases independent of chemotherapy: a single-center retrospective study in Japan
Source: Oncotarget. 2018 Mar 30;9(24):17056–65. doi: 10.18632/oncotarget.24842 (PMC5908305; doi:10.18632/oncotarget.24842)
Supplement: Supplementary file 1 [file oncotarget-09-17056-s001.pdf]

CDX2 expression is concordant between primary colorectal cancer lesions and corresponding liver metastases independent of chemotherapy: a single-center retrospective study in Japan

SUPPLEMENTARY MATERIALS

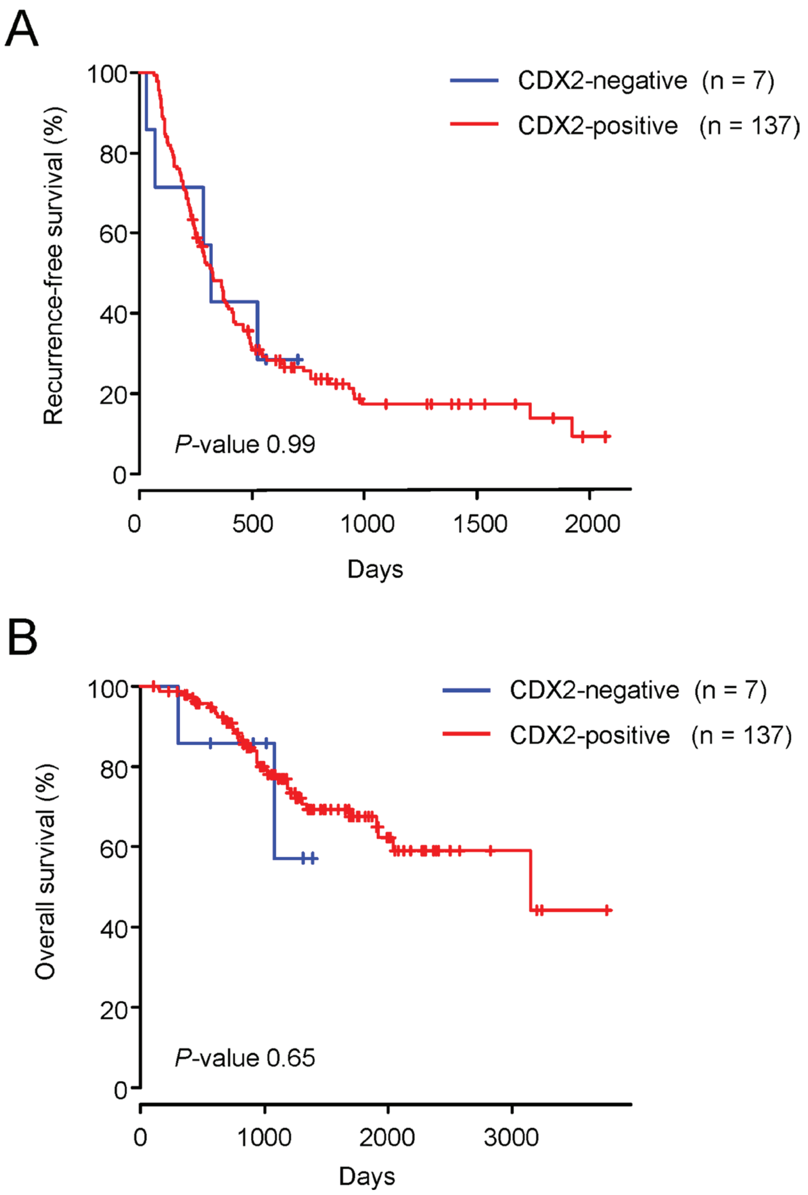

Supplementary Figure 1: (**A**, **B**) Kaplan–Meier curves of recurrence-free survival (RFS) and overall survival (OS). (A) Median RFS after liver metastasectomy in the patients with CDX2-positive CRCs was similar to that in the patients with CDX2-negative CRCs (326 days vs. 319 days, $p = 0.99$). (B) Three-year OS rate after liver metastasectomy in the patients with CDX2-positive CRCs was 78% compared with 57% in the patients with CDX2-negative CRCs ($p = 0.65$).

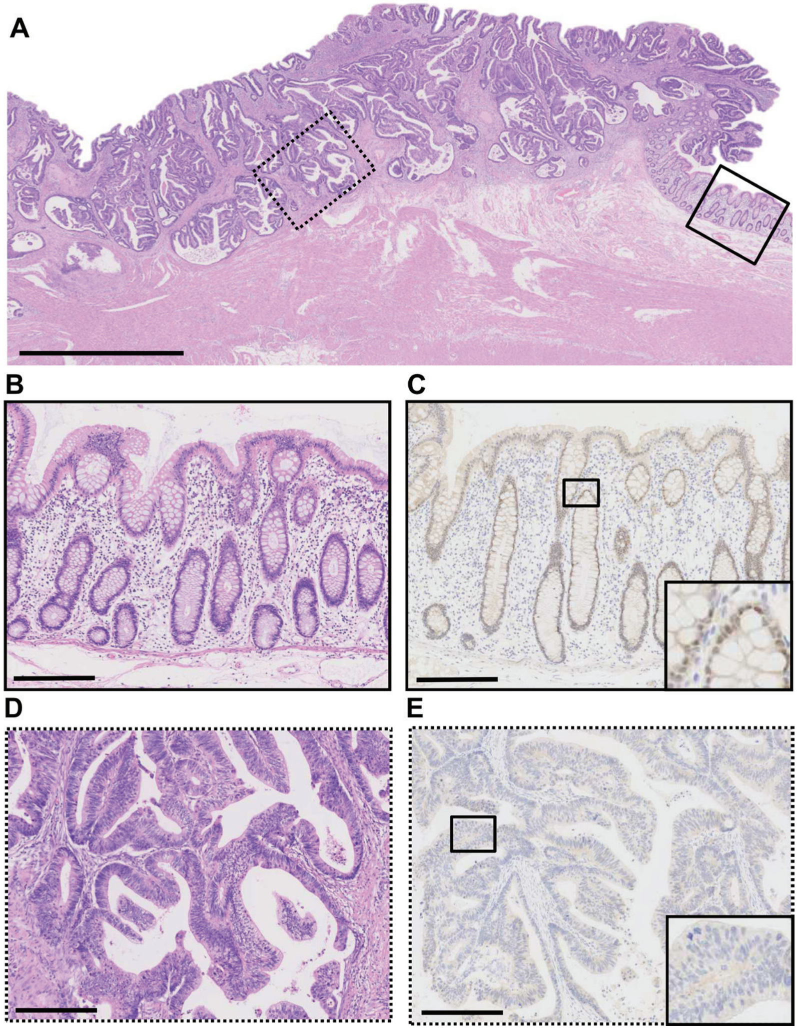

Supplementary Figure 2: CDX1 expression in a whole formalin-fixed, paraffin-embedded section of a colorectal cancer case. (**A**) A representative section of cancerous and non-cancerous tissue stained with hematoxylin and eosin. (**B**, **C**) Magnification of the area boxed by the solid line in (A). The area indicates non-cancerous tissue in the section. (B) Hematoxylin and eosin staining. (C) Immunohistochemistry (IHC) for CDX1. An inset shows a higher magnification of the boxed area. In the non-cancerous tissue, the nuclear expression of CDX1 was observed in the epithelium. (**D**, **E**) Magnification of the area boxed by the dashed line in (A). An inset shows higher magnification of the boxed area. This area indicates cancerous tissue in the section. (D) Hematoxylin and eosin staining. (E) IHC for CDX1. In the cancerous region, cancer cells do not show the nuclear CDX1 expression. Scale bars, 2.5 mm (for A) and 250 mm (for B, C, D, and E).

Supplementary Table 1: Concordance of CDX2 expression status (Cutoff point: 20%)

CDX2				
	Metastatic site			*P*-value
Primary site	Positive	Negative	Total	<0.001
Positive	109	6	115	
Negative	1	28	29	
Total	110	34	144	

Positive: <20% of CDX2-negative cancer cells.
Negative: ≥20% of CDX2-negative cancer cells.

Supplementary Table 2: Concordance of CK7, CK20, CEA, and MUC2 expression status

CK7					CK20				
	**Metastatic site**			***P*-value**		**Metastatic site**			***P*-value**
**Primary site**	**Positive**	**Negative**	**Total**	**<0.001**	**Primary site**	**Positive**	**Negative**	**Total**	**0.0016**
Positive	5	2	7		Positive	114	5	119	
Negative	2	125	127		Negative	10	5	15	
Total	7	127	134		Total	124	10	134	
**CEA**					**MUC2**				
	**Metastatic site**			***P*-value**		**Metastatic site**			***P*-value**
**Primary site**	**Positive**	**Negative**	**Total**	**0.0116**	**Primary site**	**Positive**	**Negative**	**Total**	**<0.001**
Positive	108	6	114		Positive	3	1	4	
Negative	15	5	20		Negative	1	129	130	
Total	123	11	134		Total	4	130	134	
